# Investigation of Tick-Borne Pathogens in *Ixodes ricinus* in a Peri-Urban Park in Lombardy (Italy) Reveals the Presence of Emerging Pathogens

**DOI:** 10.3390/pathogens10060732

**Published:** 2021-06-10

**Authors:** Alessandra Cafiso, Emanuela Olivieri, Anna Maria Floriano, Giulia Chiappa, Valentina Serra, Davide Sassera, Chiara Bazzocchi

**Affiliations:** 1 Department of Veterinary Medicine, University of Milan, Via dell’Università 6, 26900 Lodi, Italy; giulia.chiappa@unimi.it (G.C.); valentina.serra@unimi.it (V.S.); chiara.bazzocchi@unimi.it (C.B.); 2 Department of Biology and Biotechnology, University of Pavia, Via Ferrata 9, 27100 Pavia, Italy; emanuelaolivieri84@gmail.com (E.O.); annamaria.floriano01@universitadipavia.it (A.M.F.); davide.sassera@unipv.it (D.S.); 3 Coordinated Research Center “EpiSoMI”, University of Milan, 20133 Milan, Italy

**Keywords:** tick-borne diseases, *Neoehrlichia mikurensis*, *Ehrlichia*-like, Schotti variant, *Babesia venatorum*, EU1, *Babesia capreoli*, *Borrelia burgdorferi* s.l., tick bite, Northern Italy

## Abstract

Ticks are important vectors of a great range of pathogens of medical and veterinary importance. Lately, the spread of known tick-borne pathogens has been expanding, and novel ones have been identified as (re)emerging health threats. Updating the current knowledge on tick-borne pathogens in areas where humans and animals can be easily exposed to ticks represents a starting point for epidemiological studies and public awareness. A PCR screening for tick-borne pathogens was carried out in *Ixodes ricinus* ticks collected in a peri-urban recreational park in Ticino Valley, Italy. The presence of *Rickettsia* spp., *Borrelia burgdorferi* senso latu complex, *Anaplasma* spp. and *Babesia* spp. was evaluated in a total of 415 *I. ricinus* specimens. *Rickettsia* spp. (*R monacensis* and *R. helvetica*) were detected in 22.96% of the samples, while *B. burgdorferi* s.l. complex (*B. afzelii* and *B. lusitaniae*) were present in 10.94%. *Neoehrlichia mikurensis* (1.99%) and *Babesia venatorum* (0.73%) were reported in the area of study for the first time. This study confirmed the presence of endemic tick-borne pathogens and highlighted the presence of emerging pathogens that should be monitored especially in relation to fragile patients, the difficult diagnosis of tick-borne associated diseases and possible interactions with other tick-borne pathogens.

## 1. Introduction

Ticks are hematophagous arthropods considered, along with mosquitoes, the main vectors of important infectious diseases in humans, livestock and domestic animals worldwide [1]. In recent years, the spread of tick-borne-associated microorganisms and pathogens have been expanding [2,3]. The use of molecular biology and genomic analyses have indeed allowed the discovery of new microbial species, strains or genetic variants, increasing the number of potential health-threatening microorganisms associated with ticks (e.g., *Candidatus* Neoerhlichia mikurensis—hereafter, *N. mikurensis*—and *Babesia venatorum* [3,4,5,6,7,8,9,10]). In the context of increasing numbers of immunocompromised patients, due to the extended use of novel therapeutic approaches involving extensive immunosuppression [11], the risk of tick-borne diseases (TBDs) needs even more awareness. Symptomatic infections from *N. mikurensis* can be triggered in immunosuppressed patients, with symptoms resembling diseases such as Lyme disease (LD) [12]. Additionally, tick-borne infections through blood transfusions and organ transplantations represent a challenging issue, one that warrants the better implementation of tick-borne pathogens (TBPs) surveillance [13].

Italy is endemic for a considerable number of TBPs, and *Ixodes ricinus*, one of the most common tick species in this country, is responsible for the transmission of over 90% of the TBDs occurring in the area and in Europe [14]. Due to its triphasic behavior and low host specificity, *I. ricinus* is considered one of the primary vectors of multiple pathogens that affect human and animal health [15,16], including *Borrelia burgdorferi* sensu lato complex genospecies, spotted fever group (SFG) rickettsiae, *Anaplasma phagocytophilum*, *N. mikurensis* and *Babesia* spp. [17,18,19,20,21,22]. The distribution of *I. ricinus* in Europe has expanded during the last decades as a result of multipartite interactions between global warming, anthropogenically induced factors and changes in forest and wildlife management that, in turn, affect the available habitats for ticks [23,24]. Peri-urban recreational areas, where populations of large mammals (e.g., roe deer and wild boar) are increasing, have also become a crucial meeting point between humans, pets and ticks and currently represent a risk for the shifting of the natural transmission cycles of some TBPs [25]. Furthermore, the expansion of recreational outdoor activities has greatly increased the incidence of tick bites, leading to a higher risk of infection with TBPs [26]. Thus, the evaluation of TBPs distribution needs a constant update to maintain the awareness of tick-related diseases. The first step for TBDs surveillance should consist of assessing the diversity of pathogens occurring in a given area and their relative epidemiological importance [27]. In addition, information on coinfections in ticks is pivotal, since different combinations of pathogens in humans and animals are likely to lead to different symptoms, the varying severity of the outcoming disease and to have a negative impact on the diagnosis [28]. In the coming years, TBDs are expected to become one of the major concerns for public health in Europe [27], and the surveillance of TBPs in certain areas has become essential for epidemiological studies and risk assessment for both humans and animals.

The aim of this study was to update and evaluate the presence of both endemic and emerging TBPs of significant public health importance occurring in a Northern Italy peri-urban park and nature reserve characterized by a growing number of human outdoor activities.

## 2. Results

A total of 415 ticks were collected and all identified as *I. ricinus*. Two hundred and ninety-seven out of 415 collected specimens were nymphs (grouped in 50 pools and 47 single specimens), while the remaining 118 were adults (58 females and 60 males), for a total of 215 DNA samples. Overall, 46.98% (101/215) of the screened DNA samples were positive for at least one pathogen.

The most prevalent TBP was *Rickettsia* spp., with 22.96% positive samples (78/215; CI: 18.61–27.76). Thirty out of 78 *Rickettsia* spp. *gltA* gene amplicons were sequenced to determine the bacterial species. Of these, 29 amplicons (accession MZ068233) showed 100% identity with *Rickettsia monacensis*, while one sample (accession MZ068234) showed 100% identity with *Rickettsia helvetica* (Supplementary Figure S1a). 

The second-most common pathogen was *B. burgdorferi* s.l. complex, which showed an overall prevalence of 10.94% (42/215; CI: 8.07–14.34). Twenty out of 42 *groEL* amplicons were sequenced, resulting in a total of five *Borrelia afzelii* (accession MZ068235 and MZ068236, 98.35–100% identity with the sequences deposited in GenBank) and 15 *Borrelia lusitaniae* samples (accession MZ068237, 100% identity with the *B. lusitaniae* sequences deposited in GenBank; Supplementary Figure S1b).

*Anaplasma* spp. were detected in 1.99% of the samples screened by performing the related PCR protocol (8/215; CI: 0.91–3.67). After sequencing, all PCR products (accession MZ049694) revealed 100% identity with *N. mikurensis* (Supplementary Figure S1c).

*Babesia* spp. were detected in 0.98% of the samples (4/215; CI: 0.30–2.26), and all the obtained amplicons were subjected to sequencing. Three samples (accession MZ049960) showed 100% identity with *B. venatorum* (0.73% prevalence; CI: 0.18–1.88)*,* and one sample (accession MZ050063) showed 100% identity with *Babesia capreoli* (0.24% prevalence, CI: 0.01–1.06; Supplementary Figure S1d).

In 118 adults and 47 single nymphs, the total observed rate of coinfection was 7.27% (12/165). The coinfection prevalence was 5.93% (7/118) in adult specimens and 10.64% (5/47) in single nymph samples. In detail, 11 of the 12 samples were coinfected with SFG *Rickettsia* spp. and *B. burgdorferi* s.l. complex genospecies, while, in a nymph, a triple infection of *B. lusitaniae*, *R. monacensis* and *N. mikurensis* was observed.

## 3. Discussion

The increase of recreational outdoor activities and land usage in the last decades, as well as conservational and restocking programs for wild ungulates in nature reserves [4], have led to an increased risk for people and domestic animals to get tick bites. In this situation, a continuous monitoring on TBPs is pivotal in the context of a One Health approach. Despite Northern Italy encompassing the areas classified as endemic for important TBPs, including the *B. burgdorferi* s.l. complex [29], only fragmented investigations have been performed throughout the years [29,30,31,32]. 

The results of this study confirm the presence of endemic TBPs vectored by *I. ricinus* and also highlight the presence of emerging, previously undetected pathogens of public health concern in a Northern Italy peri-urban park close to the great urbanized area of the city of Milan and nature reserve characterized by human outdoor activities. 

The study area, close to the Ticino River, represents the optimal environment for *I. ricinus*, with a well-established biocenosis of small and large mammals, birds and reptiles. Populations of large animals such as roe deer have recently become more abundant, thanks to repopulation programs in the 1990s [33,34]. All these features represent favorable factors for the maintenance of the *I. ricinus* complete life cycle and, consequently, of the vectored pathogens.

We detected *Rickettsia* spp. at a higher prevalence (23%) compared to the other studies performed in Northern Italy on field-collected *I.* *ricinus*. The prevalence in previous reports of *Rickettsia* spp. in host-seeking ticks in Italy ranged from 1.6% and 19.23% [35]. The *Rickettsia* prevalence could be influenced by several factors, including the seasonality or year of sampling, environment, tick hosts and differences in the sensitivity of the PCR protocols [36]. The sequencing indicated *R. monacensis* as the most common species, while only one sequenced amplicon belonged to *R. helvetica*. This is consistent with the geographical distribution of the two species throughout Europe, with *R. monacensis* being more common in *I. ricinus* populations in Southern Europe [37]. *Rickettsia monacensis* is an emerging pathogen that has been shown to cause a Mediterranean spotted fever-like illness in humans in different European countries, including Italy [38]. Although the bacterium has also been detected in domestic animals, such as dogs and cats [39], no cases of clinical illness in such hosts have been reported [40]; thus, systematic approaches to address the pathogenicity of this infectious bacterial species in nonhuman patients should also be undertaken [41]. The other detected *Rickettsia* species, *R. helvetica,* was previously considered to be nonpathogenic, but it was subsequently included among the SFG rickettsiae after being associated with human illness, with infections suspected to have caused perimyocarditis, unexplained febrile illness and sarcoidosis [42]. Additionally, three cases of a mild form of human rickettsiosis were attributed to *R. helvetica* in Northern Italy through serological analyses [43]. To the best of our knowledge, no information was previously available on the presence of *R. monacensis* and *R. helvetica* in questing ticks in this study area. The closest report of these species was a screening performed on the ticks collected from migratory birds, which highlighted the presence of both *R. monacensis* and *R. helvetica* in the province of Como, Lombardy region [32]. The obtained results suggested that the bacteria belonging to this species are not occasional findings brought by migratory animals but are more likely well-established actors in the *I. ricinus*–vertebrate host interaction. 

The *Borrelia burgdorferi* s.l. complex showed an overall prevalence of 10.94%, in line with the values previously observed in Northern Italy [29,44]. Despite the presence of *B. afzelii* and *B. lusitaniae* was previously observed in a study performed in the same area, the obtained results showed *B. lusitaniae* as being the more represented, in contrast to what reported by Pistone and colleagues in 2010 [29]. It must be noted that *B. lusitaniae* is considered the most prevalent genospecies of the *B. burgdorferi* s.l. complex in ticks in Southern Europe and the Mediterranean area [45]. Moreover, lizards are the most likely reservoirs for *B. lusitaniae* [46,47,48] and are known to be present in the area with high *I. ricinus* infestation rates [33]. Conversely, *B. afzelii* is maintained by rodents [49], which are reservoirs for this *B. burgdorferi* s.l. complex genospecies and are widespread in the study area [50,51]. *Borrelia afzelii* is one the most common *B. burgdorferi* s.l. complex genospecies, together with *B. garinii*, and infects ticks with the highest prevalence rates in the Central European countries [44]. 

No samples were found positive for *Anaplasma* spp. This result is in contrast with the reports on this bacterial genus performed in Italy [9,20,27,52], although scarce information about this pathogen in the questing *I. ricinus* is available for the Lombardy region. Future studies should be focused on the investigation of *Anaplasma phagocytophilum* in ticks and blood samples recovered from roe deer or wild boar (which have been demonstrated to be natural hosts for this pathogen [20]). *Neoehrlichia mikurensis*, another member of the *Anaplasmataceae* family, was found in the area of study. Although *N. mikurensis* has been reported with a prevalence between 1% and over 20% throughout Europe [53], and previous surveys have highlighted its presence in Northeastern Italy [54], to the best of our knowledge, the present work represents the first detection of this emerging TBP in Northwestern Italy. *Neoehrlichia mikurensis* has been raising attention for causing symptoms resembling those of LD, including erythema migrans (EM)-like rashes [12,55], leading to *N. mikurensis* infection misdiagnoses [56]. Since LD is relatively common in Northern Italy [29,57], the cooccurrence of *N. mikurensis* warrants deeper investigations and population’s awareness. Furthermore, a *N. mikurensis* infection may show similarities to *Anaplasma* or *Ehrlichia* infections [58], as neoehrlichiosis displays clinical signs that may vary in severity but are usually nonspecific, such as a fever, lethargy, myalgia, arthralgia and anorexia [53]. This should also be taken into consideration in light of coinfections in tick vectors, as observed in one of the screened samples. Since no serological test for *N. mikurensis* is currently available [54], molecular screenings of reservoir hosts, particularly small mammals such as rodents of the genera *Apodemus* spp. or *Microtus* spp. [59], could provide important information on the circulation of the pathogen. 

*Babesia venatorum* (formerly known as *Babesia* sp. EU1), whose primary host is considered to be roe deer, was previously identified in ticks from the region of Northern Italy with similar low infection rates as those observed in this study [27,35,52]. Notably, *B. venatorum* can cause clinical manifestations of different severity in immunocompromised or splenectomized humans [5,60]. Nevertheless, it is estimated that *B. venatorum* infections may have been overlooked or misdiagnosed, possibly due to serologic cross-reactivity in laboratory diagnostic tests [61]. *Babesia capreoli*, a species closely related to *Babesia divergens*, is typically found to infect roe deer but seems to be apathogenic for sheep, cattle and humans [62,63]. *Babesia capreoli* was previously reported in the blood and ticks collected from roe deer in Italy [64,65].

Additionally, attention should be paid to coinfections, since hosts coinfected with different pathogens may show more severe symptoms of diseases [66]. A positive association between *B. burgdorferi* s.l. complex genospecies and *Rickettsia* spp. has been observed in several studies [66,67], possibly resulting in higher replication rates of the two species both in the vector and the vertebrate host [68]. 

Considering the increasing role of peri-urban areas in recreational activities, the presence of TBPs represents a red flag for animal and human health that should be constantly monitored. Emerging pathogens such as *N. mikurensis* and *B. venatorum* should be kept under surveillance, especially in light of infections in fragile patients [69], and should be considered in cases where the etiological agents of commonly known TBDs are not detected.

## 4. Materials and Methods

### 4.1. Collection Site

Ticks were collected in the “La Fagiana” Nature Reserve (Pontevecchio, 45°26′07.1″ N 8°49′46.7″ E), within the central area of Ticino Valley Nature Park (Lombardy region, Northern Italy), 40 km from the city of Milan. The 500-ha peri-urban area, around 134 m.a.s.l., is characterized by forest areas, meadows, oxbow lakes and wetlands and represents the most important public nature reserve in Ticino Valley. The forest is normally used for leisure, educational activities and dog-walking, and represents an important site for wildlife. The fauna consists of small- and medium-sized mammals (e.g., roe deer and wild boar); birds and small reptiles, such as lizards. 

### 4.2. Tick Collection

Host-seeking ticks were collected in 2019 by the unsystematic flagging and dragging of low vegetation during the period of high seasonal activity of *I. ricinus* in Central Europe (between April and October) using a 1 m^2^ woolen blanket. Samplings were performed close to pathways and picnic areas, where humans and domestic animals are more likely to be exposed to ticks during outdoor activities.

Once collected, ticks were grouped according to their sex and developmental stage and preserved in 70% ethanol. Subsequently, ticks were identified on the basis of their morphological features [70], and nymphs were grouped in pools of 1–5 specimens for a total of 97 nymph samples, while the 118 collected adults were treated individually; the specimens were stored at +4 °C until further analyses.

All the specimens (118 adults and 97 nymph samples, for a total of 215 samples) were subsequently processed for DNA extraction and the molecular detection of TBPs.

### 4.3. Molecular Analyses

DNA was extracted from single adult ticks and pools of nymphs using the DNeasy Blood and Tissue Kit (Qiagen, Hilden, Germany) following the manufacturer’s instructions. Extracted DNA samples were quantified and stored at −80 °C for subsequent analyses. Quality of the extracted DNA was assessed by amplifying a fragment of ~360 bp of the 12S rDNA of Ixodidae [71]. The DNA samples were then tested with specific qualitative PCR protocols designed for the amplification of SFG rickettsiae, *Anaplasma* spp., *B. burgdorferi* s.l. and *Babesia* spp. DNA. For *Babesia divergens*/*B. capreoli* species differentiation, a de novo reverse primer was designed to amplify a ~1200-bp portion of the 18S rDNA gene (all target genes, primers and references are reported in Table 1). According to Malandrin et al. [62], the three base differences in the 18S rDNA amplified fragment (positions 631, 663 and 1637, with AAC for *B. divergens* and GTT for *B. capreoli*) can discriminate the two species.

The obtained PCR products were excised from agarose gel and purified using the Wizard^®^ SV Gel and PCR Clean-Up System Kit (Promega, Madison, WI, USA) according to the manufacturer’s protocols and bidirectionally Sanger-sequenced. Sequences were then assembled, manually curated with SeaView 4.7 [76] and compared with the representative sequences available in NCBI GenBank using BLAST. The obtained sequences were deposited in GenBank.

### 4.4. Phylogenetic and Statistical Analyses 

The prevalence of each pathogen was calculated with the estimated pooled prevalence (EPP) with a 95% confidence interval (95% CI) using the online pool prevalence calculator Epitools [77]. The method estimates the prevalence and confidence limits for variable pool sizes and assumes 100% test sensitivity and specificity. 

All the phylogenetic inferences were performed as follows: sequences were aligned with MUSCLE v3.8.31 [78], the evolutionary model to be used for phylogenetic inference was chosen according to the AIC (using modeltest-ng [79]) and the phylogeny was inferred using RAxML 8.2.4 (100 bootstraps, -p 123, -x 1234 [80]). The evolutionary models applied for phylogenetic inference were: GTR+I+G for *Babesia* spp. and *Anaplasma* spp./*Neoehrlichia* spp., HKY+I+G for the *B. burgdorferi* s.l. complex genospecies and *Rickettsia* spp.

## Figures and Tables

**Table 1:** List of tick-borne pathogens (TBPs), target genes, PCR primer names and nucleotide sequences and references of the PCR primers used in the TBP screening.

TBP	Target Gene	Primer Name	Nucleotide Sequence (5′-3′)	Reference
SFG rickettsiae	*gltA*	Rp877p	GGGGACCTGCTCACGGCGG	[72]
		Rp1258n	ATTGCAAAAAGTACAGTGAACA	
*Anaplasma* spp.	16S rRNA	16S8FE	GGAATTCAGAGTTGGATCATGGCTCAG	[73]
		B-GA1B_mod	CGGGATCCCGAGTTTGCCGGGACTT ^1^	
*B. burgdorferi* s.l.	*groEL*	groEL-F	ACGATTTCTTATGTTGAGGG	[74]
		groEL-R	TCTCAAGAACTGGTAAAAG	
*Babesia* spp.	18S rDNA	PIRO-A	AATACCCAATCCTGACACAGGG	[75] ^2^
		PIRO-B	TTAAATACGAATGCCCCCAAC	
*B. divergens*/ *B. capreoli* ^3^	18S rDNA	PIRO-A	AATACCCAATCCTGACACAGGG	[75]
		Piro-900b	AACCTTGTTACGACTTCTCC	This work

^1^ Modified by the authors. ^2^ PCR annealing step performed at 62 °C. ^3^ PCR protocol and conditions: 95 °C for 3 min; 5 cycles at 95 °C for 30 s, 64 °C for 30 s and 72 °C for 60 s; 15 cycles at 95 °C for 30 s, 60 °C for 30 s and 72 °C for 60 s; 20 cycles at 95 °C for 30 s, 54 °C for 30 s and 72 °C for 60 s and final elongation 72 °C for 5 min. Amplicon size ~1200 bp.

## Data Availability

The obtained gene sequences were deposited in the NCBI GenBank database. The accession numbers of the obtained sequences are contained within the article and in the Supplementary Materials.

## Supplementary Materials

The following are available online at https://www.mdpi.com/article/10.3390/pathogens10060732/s1: Figure S1: Phylogenetic inferences of the obtained gene sequences.
