# Supplementary material for: Investigation of Tick-Borne Pathogens in Ixodes ricinus in a Peri-Urban Park in Lombardy (Italy) Reveals the Presence of Emerging Pathogens
Source: Pathogens. 2021 Jun 10;10(6):732. doi: 10.3390/pathogens10060732 (PMC8230559; doi:10.3390/pathogens10060732)
Supplement: Supplementary file 1 [file pathogens-10-00732-s001.zip › pathogens-1241441-supplementary.pdf]

Figure S1

Phylogenetic inference of the obtained gene sequences. Phylogenetic inference performed on the obtained gene sequences for the following tick-borne pathogens: a) Spotted Fever Group *Rickettsia* spp., b) *Borrelia burgdorferi* s.l., c) *Anaplasma* spp./*Neoehrlichia spp.*, d) *Babesia* spp. Phylogenetic inferences were performed using RAxML 8.2.4 (100 bootstraps, -p 123, -x 1234). The evolutionary models applied for phylogenetic inference were: GTR+I+G for *Babesia* spp. and *Anaplasma* spp./*Neoehrlichia* spp., HKY+I+G for *Borrelia* spp., and *Rickettsia* spp. The species detected in this work are indicated in red font, GenBank accession numbers are found next to the species names.

a)

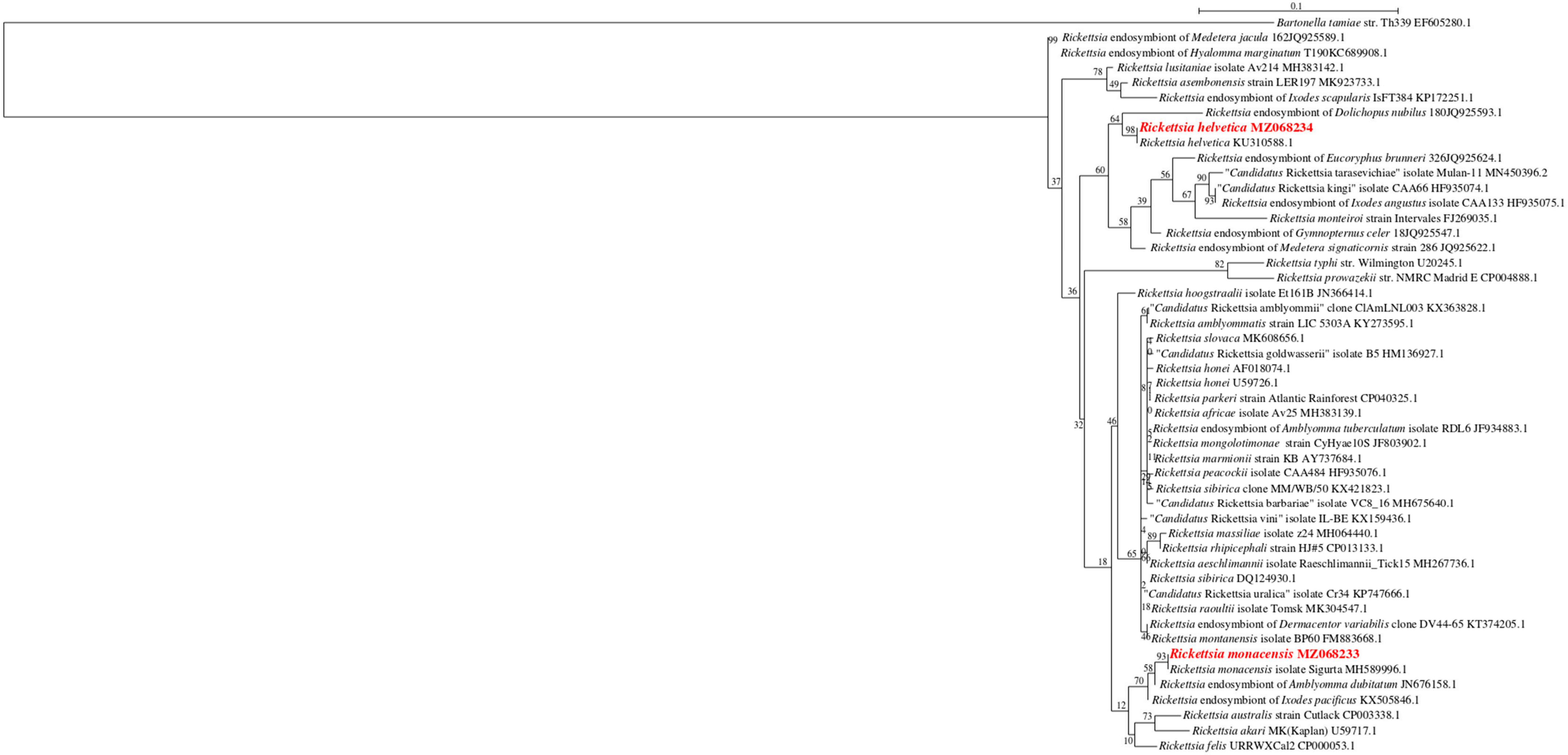
0.1
Bartonella tamiae str. Th339 EF605280.1
Rickettsia endosymbiont of Medetera jacula 162JQ925589.1
Rickettsia endosymbiont of Hyalomma marginatum T190KC689908.1
Rickettsia lusitaniae isolate Av214 MH383142.1
Rickettsia asembonensis strain LER197 MK923733.1
Rickettsia endosymbiont of Ixodes scapularis IsFT384 KP172251.1
Rickettsia endosymbiont of Dolichopus nubilus 180JQ925593.1
Rickettsia helvetica MZ068234
Rickettsia helvetica KU310588.1
Rickettsia endosymbiont of Eucoryphus brunneri 326JQ925624.1
"Candidatus Rickettsia tarasevichiae" isolate Mulan-11 MN450396.2
"Candidatus Rickettsia kingi" isolate CAA66 HF935074.1
Rickettsia endosymbiont of Ixodes angustus isolate CAA133 HF935075.1
Rickettsia monteiroi strain Intervales FJ269035.1
Rickettsia endosymbiont of Gymnopternus celer 18JQ925547.1
Rickettsia endosymbiont of Medetera signaticornis strain 286 JQ925622.1
Rickettsia typhi str. Wilmington U20245.1
Rickettsia prowazekii str. NMRC Madrid E CP004888.1
Rickettsia hoogstraalii isolate Et161B JN366414.1
"Candidatus Rickettsia amblyommii" clone ClAmLNL003 KX363828.1
Rickettsia amblyommatis strain LIC 5303A KY273595.1
Rickettsia slovaca MK608656.1
"Candidatus Rickettsia goldwasserii" isolate B5 HM136927.1
Rickettsia honei AF018074.1
Rickettsia honei U59726.1
Rickettsia parkeri strain Atlantic Rainforest CP040325.1
Rickettsia africae isolate Av25 MH383139.1
Rickettsia endosymbiont of Amblyomma tuberculatum isolate RDL6 JF934883.1
Rickettsia mongolotimonae strain CyHyae10S JF803902.1
Rickettsia marmionii strain KB AY737684.1
Rickettsia peacockii isolate CAA484 HF935076.1
Rickettsia sibirica clone MM/WB/50 KX421823.1
"Candidatus Rickettsia barbariae" isolate VC8_16 MH675640.1
"Candidatus Rickettsia vini" isolate IL-BE KX159436.1
Rickettsia massiliae isolate z24 MH064440.1
Rickettsia rhipicephali strain HJ#5 CP013133.1
Rickettsia aeschlimannii isolate Raeschlimannii_Tick15 MH267736.1
Rickettsia sibirica DQ124930.1
"Candidatus Rickettsia uralica" isolate Cr34 KP747666.1
Rickettsia raoultii isolate Tomsk MK304547.1
Rickettsia endosymbiont of Dermacentor variabilis clone DV44-65 KT374205.1
Rickettsia montanensis isolate BP60 FM883668.1
Rickettsia monacensis MZ068233
Rickettsia monacensis isolate Sigurta MH589996.1
Rickettsia endosymbiont of Amblyomma dubitatum JN676158.1
Rickettsia endosymbiont of Ixodes pacificus KX505846.1
Rickettsia australis strain Cutlack CP003338.1
Rickettsia akari MK(Kaplan) U59717.1
Rickettsia felis URRWXCal2 CP000053.1

b)

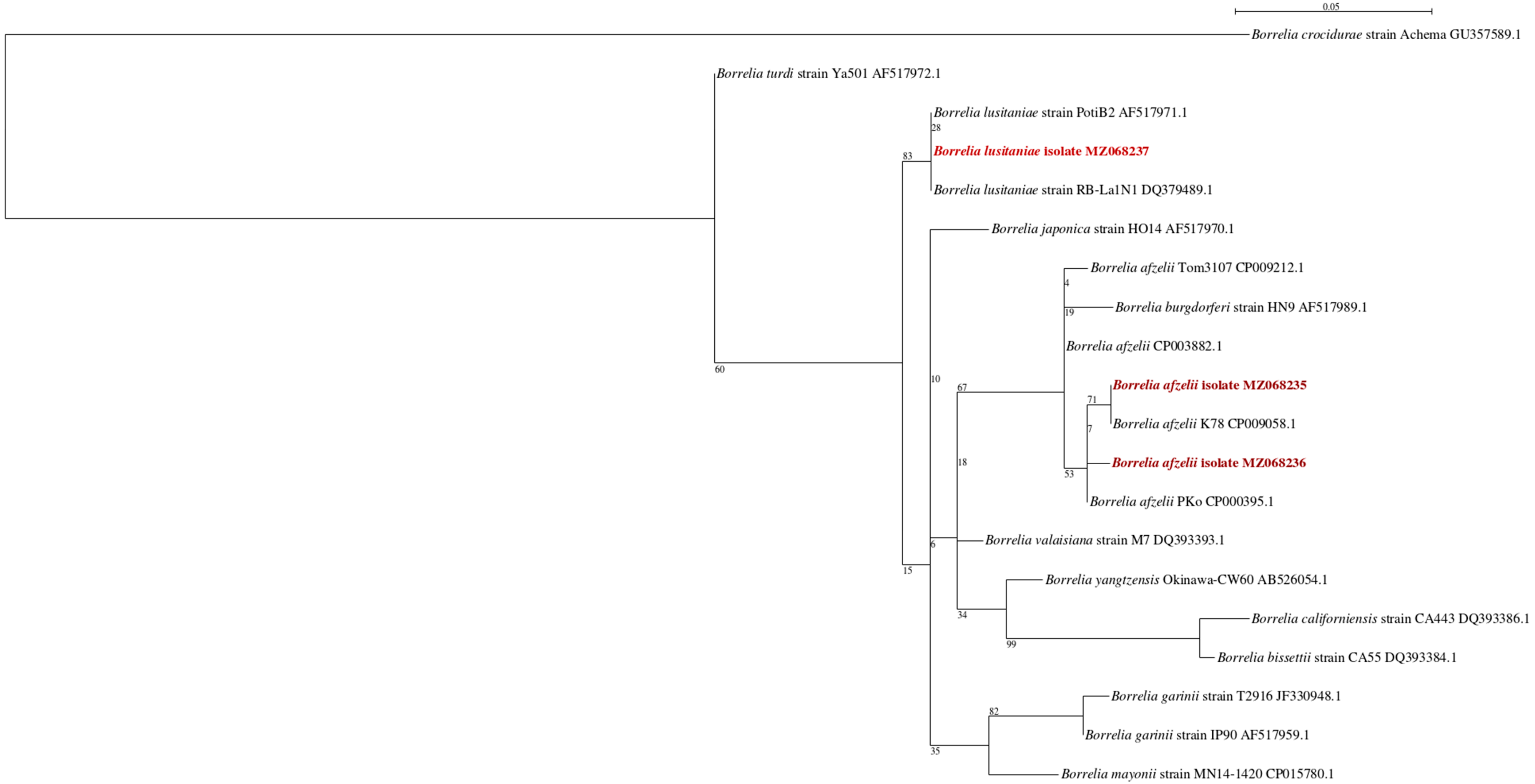
0.05
Borrelia crocidurae strain Achema GU357589.1
Borrelia turdi strain Ya501 AF517972.1
Borrelia lusitaniae strain PotiB2 AF517971.1
Borrelia lusitaniae isolate MZ068237
Borrelia lusitaniae strain RB-La1N1 DQ379489.1
Borrelia japonica strain HO14 AF517970.1
Borrelia afzelii Tom3107 CP009212.1
Borrelia burgdorferi strain HN9 AF517989.1
Borrelia afzelii CP003882.1
Borrelia afzelii isolate MZ068235
Borrelia afzelii K78 CP009058.1
Borrelia afzelii isolate MZ068236
Borrelia afzelii PKo CP000395.1
Borrelia valaisiana strain M7 DQ393393.1
Borrelia yangtzensis Okinawa-CW60 AB526054.1
Borrelia californiensis strain CA443 DQ393386.1
Borrelia bissettii strain CA55 DQ393384.1
Borrelia garinii strain T2916 JF330948.1
Borrelia garinii strain IP90 AF517959.1
Borrelia mayonii strain MN14-1420 CP015780.1
28
83
60
4
19
10
67
71
7
18
53
6
15
34
99
82
35

c)

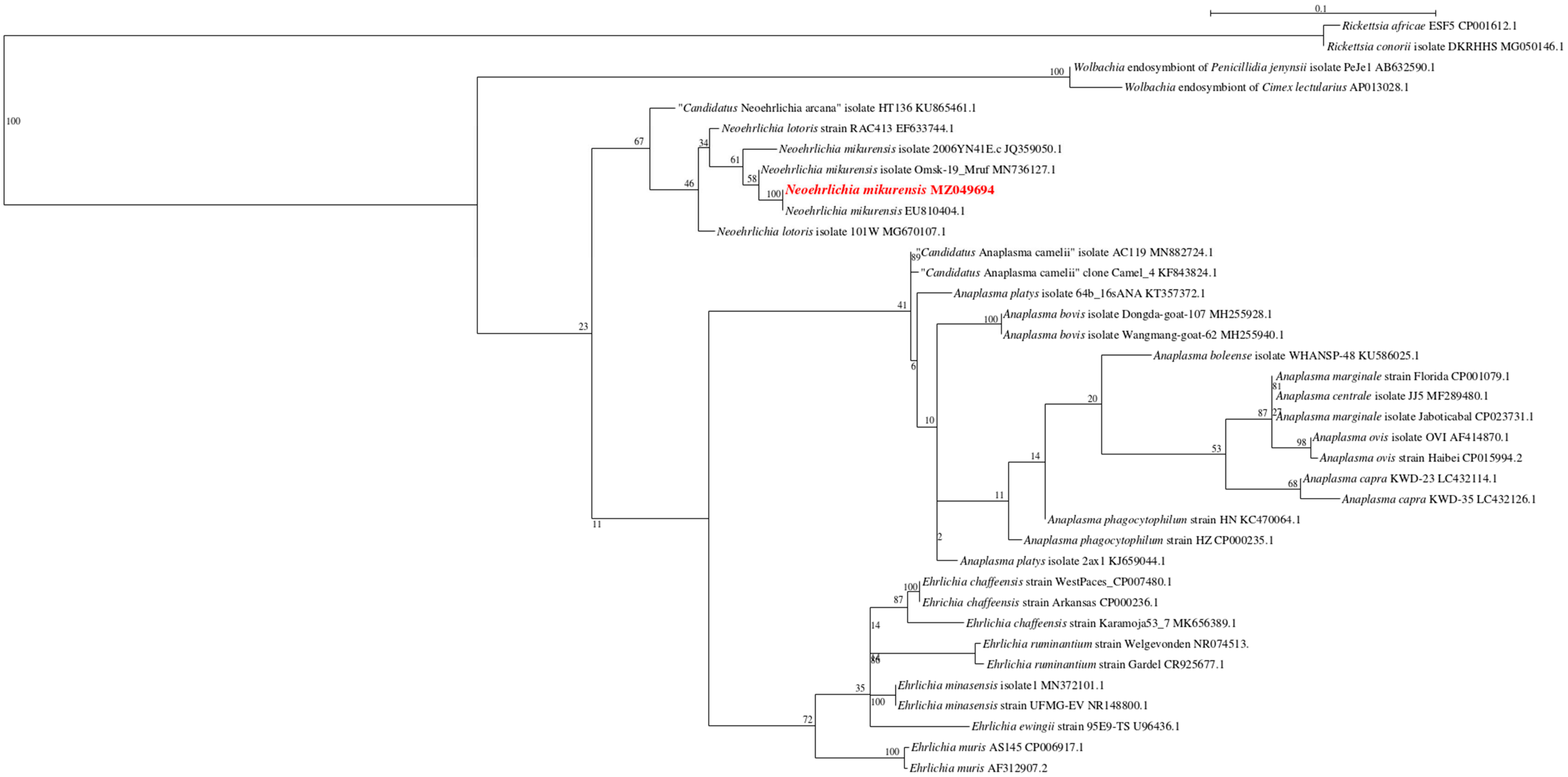
0.1
Rickettsia africae ESF5 CP001612.1
Rickettsia conorii isolate DKRHHS MG050146.1
Wolbachia endosymbiont of Penicillidia jenynsii isolate PeJe1 AB632590.1
Wolbachia endosymbiont of Cimex lectularius AP013028.1
"Candidatus Neoehrlichia arcana" isolate HT136 KU865461.1
Neoehrlichia lotoris strain RAC413 EF633744.1
Neoehrlichia mikurensis isolate 2006YN41E.c JQ359050.1
Neoehrlichia mikurensis isolate Omsk-19_Mruf MN736127.1
Neoehrlichia mikurensis MZ049694
Neoehrlichia mikurensis EU810404.1
Neoehrlichia lotoris isolate 101W MG670107.1
"Candidatus Anaplasma camelii" isolate AC119 MN882724.1
"Candidatus Anaplasma camelii" clone Camel_4 KF843824.1
Anaplasma platys isolate 64b_16sANA KT357372.1
Anaplasma bovis isolate Dongda-goat-107 MH255928.1
Anaplasma bovis isolate Wangmang-goat-62 MH255940.1
Anaplasma boleense isolate WHANSP-48 KU586025.1
Anaplasma marginale strain Florida CP001079.1
Anaplasma centrale isolate JJ5 MF289480.1
Anaplasma marginale isolate Jaboticabal CP023731.1
Anaplasma ovis isolate OVI AF414870.1
Anaplasma ovis strain Haibei CP015994.2
Anaplasma capra KWD-23 LC432114.1
Anaplasma capra KWD-35 LC432126.1
Anaplasma phagocytophilum strain HN KC470064.1
Anaplasma phagocytophilum strain HZ CP000235.1
Anaplasma platys isolate 2ax1 KJ659044.1
Ehrlichia chaffeensis strain WestPaces_CP007480.1
Ehrichia chaffeensis strain Arkansas CP000236.1
Ehrlichia chaffeensis strain Karamoja53_7 MK656389.1
Ehrlichia ruminantium strain Welgevonden NR074513.
Ehrlichia ruminantium strain Gardel CR925677.1
Ehrlichia minasensis isolate1 MN372101.1
Ehrlichia minasensis strain UFMG-EV NR148800.1
Ehrlichia ewingii strain 95E9-TS U96436.1
Ehrlichia muris AS145 CP006917.1
Ehrlichia muris AF312907.2

d)

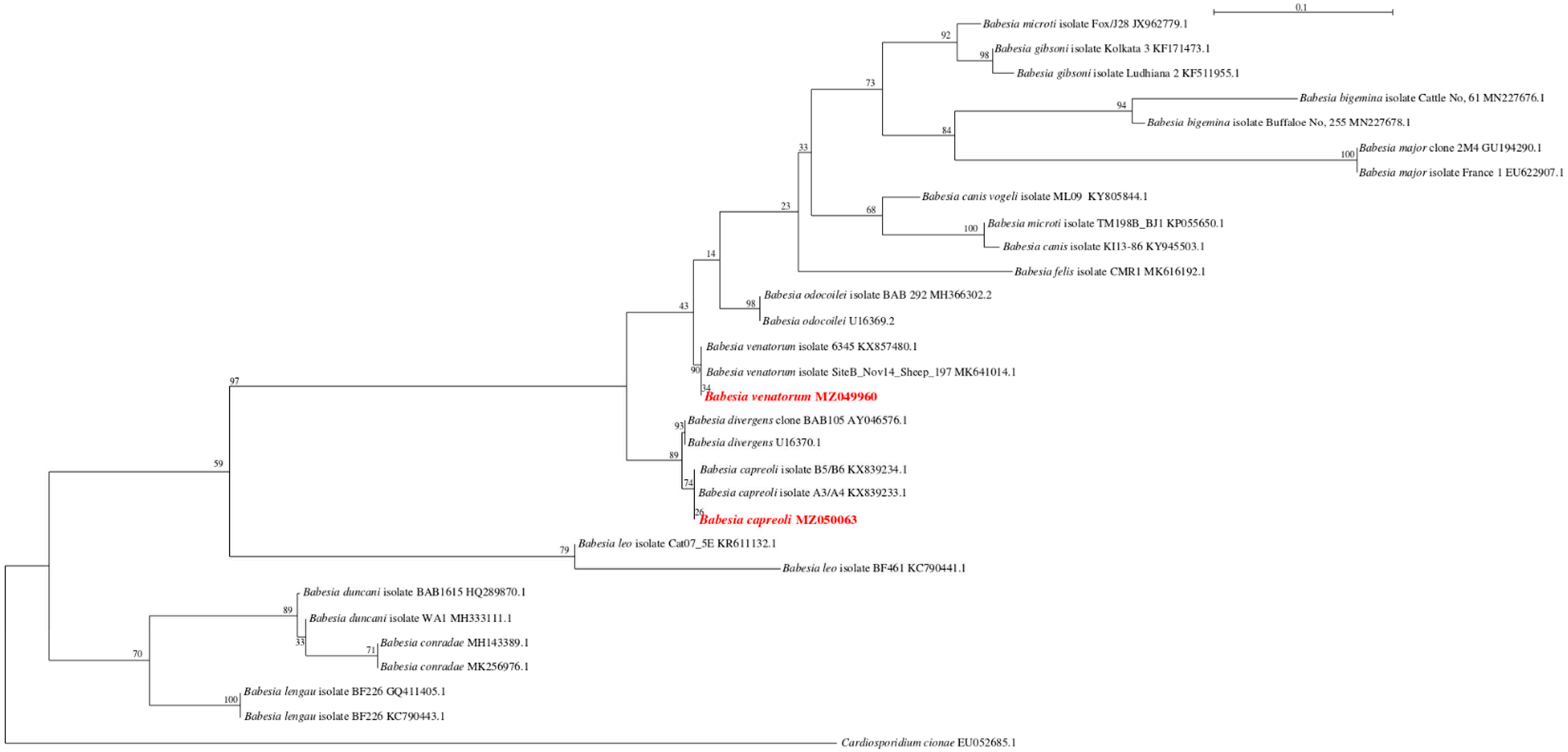
0.1
Babesia microti isolate Fox/J28 JX962779.1
Babesia gibsoni isolate Kolkata 3 KF171473.1
Babesia gibsoni isolate Ludhiana 2 KF511955.1
Babesia bigemina isolate Cattle No, 61 MN227676.1
Babesia bigemina isolate Buffaloe No, 255 MN227678.1
Babesia major clone 2M4 GU194290.1
Babesia major isolate France 1 EU622907.1
Babesia canis vogeli isolate ML09 KY805844.1
Babesia microti isolate TM198B_BJ1 KP055650.1
Babesia canis isolate KI13-86 KY945503.1
Babesia felis isolate CMR1 MK616192.1
Babesia odocoilei isolate BAB 292 MH366302.2
Babesia odocoilei U16369.2
Babesia venatorum isolate 6345 KX857480.1
Babesia venatorum isolate SiteB_Nov14_Sheep_197 MK641014.1
Babesia venatorum MZ049960
Babesia divergens clone BAB105 AY046576.1
Babesia divergens U16370.1
Babesia capreoli isolate B5/B6 KX839234.1
Babesia capreoli isolate A3/A4 KX839233.1
Babesia capreoli MZ050063
Babesia leo isolate Cat07_5E KR611132.1
Babesia leo isolate BF461 KC790441.1
Babesia duncani isolate BAB1615 HQ289870.1
Babesia duncani isolate WA1 MH333111.1
Babesia conradae MH143389.1
Babesia conradae MK256976.1
Babesia lengau isolate BF226 GQ411405.1
Babesia lengau isolate BF226 KC790443.1
Cardiosporidium cionae EU052685.1
92
98
73
94
84
100
33
23
68
100
14
98
43
90
34
97
93
89
74
26
59
79
89
33
71
70
100
